# Association between serum hydrogen sulfide concentrations and dysglycemia: a population-based study

**DOI:** 10.1186/s12902-022-00995-8

**Published:** 2022-03-28

**Authors:** Zahra Bahadoran, Sajad Jeddi, Parvin Mirmiran, Khosrow Kashfi, Fereidoun Azizi, Asghar Ghasemi

**Affiliations:** 1 Nutrition and Endocrine Research Center, Research Institute for Endocrine Sciences, Shahid Beheshti University of Medical Sciences, Tehran, Iran; 2 Endocrine Physiology Research Center, Research Institute for Endocrine Sciences, Shahid Beheshti University of Medical Sciences, No. 24, Sahid-Erabi St, Yemen St, Chamran Exp, P.O.Box: 19395-4763, Tehran, Iran; 3 Department of Clinical Nutrition and Human Dietetics, Faculty of Nutrition Sciences and Food Technology, National Nutrition and Food Technology Research Institute, Shahid Beheshti University of Medical Sciences, Tehran, Iran; 4 Department of Molecular, Cellular, and Biomedical Sciences, Sophie Davis School of Biomedical Education, City University of New York School of Medicine, New York, NY 10031 USA; 5 Endocrine Research Center, Research Institute for Endocrine Sciences, Shahid Beheshti University of Medical Sciences, Tehran, Iran

**Keywords:** Hydrogen sulfide, Type 2 diabetes, Impaired fasting glucose, Impaired glucose tolerance

## Abstract

**Background and aim:**

Hydrogen sulfide (H_2_S), a signaling gasotransmitter, is involved in carbohydrate metabolism. Here, we aimed to assess the potential association between serum H_2_S and dysglycemia in the framework of a population-based study.

**Methods:**

Adults men and women with completed data (*n* = 798), who participated in the Tehran Lipid and Glucose Study (2014–2017) were included in the study. Medians of fasting serum H_2_S concentration were compared across the glycemic status of the participants, defined as type 2 diabetes mellitus (T2DM), isolated impaired fasting glucose (IIFG), isolated impaired glucose tolerance (IIGT), combined IFG-IGT, and normal glycemia [i.e., those with both normal fasting glucose (NFG) and normal glucose tolerance (NGT)]. Multinomial logistic regression was used to assess potential associations between serum H_2_S and the defined glycemic status.

**Results:**

Mean age of the participants was 45.1 ± 14.0 y, and 48.1% were men. Prevalence of T2DM, IIFG, IIGT, and combined IFG-IGT was 13.9, 9.1, 8.1, and 4.8% respectively. No significant difference was observed in serum H_2_S concentrations between the groups. Lower serum H_2_S (< 39.6 µmol/L) was associated with an increased chance of having IIGT (OR = 1.96, 95% CI = 1.15–3.34) in the adjusted model.

**Conclusion:**

Reduced serum H_2_S level may be associated with impaired glucose tolerance.

**Supplementary Information:**

The online version contains supplementary material available at 10.1186/s12902-022-00995-8.

## Introduction

Hydrogen sulfide (H_2_S) is a signaling gasotransmitter with cytoprotective properties that has several physiological functions in the cardiovascular, neuronal, gastrointestinal, respiratory, and reproductive systems [1]. Hydrogen sulfide regulates neurotransmission, vascular tone, angiogenesis, cellular redox homeostasis [2, 3]; has an essential role in regulating cell growth and differentiation, mitochondrial biogenesis, adipose tissue metabolism; and is involved in inflammatory pathways [1, 4].

Hydrogen sulfide is endogenously synthesized in tissues that are involved in carbohydrate metabolism, i.e., pancreatic β-cells, liver, adipose tissue, skeletal muscle, and hypothalamus, thus regulating local and systemic glucose metabolism [5, 6]. In the liver, H_2_S regulates glucose uptake, glycogen storage, gluconeogenesis, and mitochondrial function [7–9]. The role of H_2_S in regulating pancreatic β-cell apoptosis and insulin secretion has remained inconclusive, and its effects within the β-cells seem to depend on the stage and type of diabetes [5, 6, 10]. Both inhibitory and stimulatory H_2_S effects on insulin-induced glucose uptake and a dual role in the development of insulin resistance have been illustrated [11–13].

Likewise, the potential role of H_2_S in the pathophysiology of type 2 diabetes mellitus (T2DM) is controversial; endogenous H_2_S synthesis has been reported to be decreased during obesity development, T2DM, and its complications [10, 14]. In addition, available evidence indicates that plasma H_2_S levels are decreased in patients with T2DM [14, 15].

The aim of this study is to determine association between fasting serum H_2_S levels and glycemic status in a population-based study.

## Methods

### Study population

**Fig. 1:**
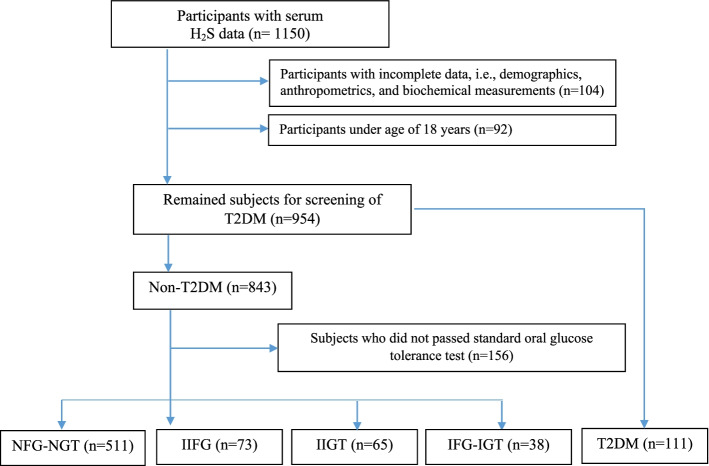
Flowchart of the study participants. T2DM, type 2 diabetes mellitus; IFG, impaired glucose tolerance; IGT, impaired glucose tolerance; IIFG, isolated IFG; IIGT, isolated IGT; NFG-NGT, normal fasting glucose-normal glucose tolerance

This cross-sectional study was conducted among the participants of an ongoing community-based prospective study (the Tehran Lipid and Glucose Study, TLGS), which started in 1999 with 15,005 individuals, aged ≥ 3 years, to investigate and prevent non-communicable diseases [16]. For the current study, we recruited a sub-set of the participants comprising the sixth phase of the TLGS (2014–2017) to measure their serum H_2_S concentrations (*n* = 1150). After exclusion of participants with incomplete data on demographics, anthropometrics, and biochemical measurements (*n* = 104) and those who were under age of 18 y (*n* = 92), 954 adult men and women remained for screening of T2DM subjects. Finally, subjects who did not passed standard oral glucose tolerance test (OGTT) were excluded (*n* = 156), and final analyses were conducted on 798 participants. The flowchart of the study participants is presented as Fig. 1.

Written informed consent was obtained from all participants. The ethics research council of the Research Institute for Endocrine Sciences, Shahid Beheshti University of Medical Sciences, Tehran, Iran, approved the study protocol (Ethics code: IR.SBMU.ENDOCRINE.REC.1400.074).

### Demographic, anthropometric, and biochemical measurements

Details of data collection and measurements of the variables in the TLGS have been reported elsewhere [16]. In brief, anthropometric data, including body weight, height, and waist circumference (WC) were collected using standard methods. Body mass index (BMI) was calculated as weight (kg) divided by square of height in meters (m^2^).

Systolic (SBP) and diastolic (DBP) blood pressures were measured using a standard mercury sphygmomanometer calibrated by the Institute of Standards and Industrial Research of Iran [17]. Blood pressure was measured twice on participants’ right arm, after a 15-min rest in a sitting position, with at least a 30-s interval between two measurements. The mean of the two measurements was considered as the participant’s blood pressure.

Details of biochemical measurements in the TLGS samples have been described elsewhere [18]. In brief, measurements of fasting serum glucose (FSG), triglyceride (TG), and high-density lipoprotein cholesterol (HDL-C) levels were all done after a 12-to 14-h overnight fast. The standard oral glucose tolerance test (OGTT) was performed for all participants who were not on glucose-lowering medications.

Total serum sulfide levels were measured using the methylene blue method [19]. This is a spectrophotometric method based on methylene blue dye after the reaction of the sulfide and N, N-dimethyl-*p*-phenylenediamine [20]. In brief, serum (100 µL) was added to a test tube containing zinc acetate (1% *w*/*v*, 200 µL), *N*,*N*-dimethyl-*p*-phenylenediamine sulfate in 7.2 M HCl (20 mM, 100 µL), and FeCl_3_ in 1.2 M HCl (30 mM, 133 µL). After incubation at 37 °C for 30 min, the tubes were centrifuged at 5000 g for 10 min. Supernatants were collected for the measurement of total sulfide. Total sulfide concentration was determined in the samples using a standard calibration curve (Supplementary Figure 1) established by 0–200 µM of sodium hydrosulfide (NaSH); optical density was read at wavelength of 670 nm using a microplate reader (BioTek, MQX2000R2, Winooski, VT, USA) [21]. Both intra- and inter-assay coefficients of variation (CV) were between 1.7–6.3%.

### Definition of terms

Participants were categorized into different groups of glycemic status as follows [22, 23]: normal glycemia [i.e., normal fasting glucose (NFG) and normal glucose tolerance (NGT)], FSG < 100 and 2 hours serum glucose (2 h-SG) < 140; isolated impaired fasting glucose (IIFG), 100 ≤ FSG < 126 and 2 h-SG < 140 mg/dL; isolated impaired glucose tolerance (IIGT), 140 ≤ 2 h-SG < 200 and FSG < 100 mg/dL; combined IFG and IGT (IFG-IGT) was defined as having both 100 ≤ FSG < 126 and 140 ≤ 2 h-SG < 200 mg/dL; T2DM, FSG ≥ 126 mg/dL or 2 h-SG ≥ 200 mg/dL, or using glucose-lowering medications.

### Statistical methods

Statistical analyses were conducted using the SPSS for Windows version 20 (SPSS Inc., Chicago, IL, USA) and the GraphPad Prism version 6.00 for Windows (GraphPad Software, CA, USA). Serum H_2_S (due to a non-normal distribution, log-H_2_S was included in the model) concentration were compared across the groups using analysis of covariance with adjustment of age and sex.

Association between serum H_2_S and glycemic status was assessed using multinomial logistic regression analysis with subjects’ glycemia status (NFG-NGT as reference, IIFG, IIGT, combined IFG-IGT, and T2DM) as the outcome variables and serum H_2_S as the independent variable [either as continues (log_10_-H_2_S) or categorical variable (< or ≥ median ≈39.6 µmol/L)]. Potential covariates were selected based on both statistical and scientific evidence. A univariate analysis was performed for potential confounding variables, and those with *P*_E_ < 0.2 were selected for the final multivariable model; *P*_E_ (*P*-value for entry) determines which variables should be included in the multivariable model [24]. Finally, two models, including crude and adjusted model (sex and subjects’ age) were conducted.

## Results

The mean age of the participants was 45.1 ± 14.0 y, and 48.1% were men. Prevalence of T2DM, IIFG, IIGT, and combined IFG-IGT was 13.9%, 9.1%, 8.1%, 4.8%, respectively. The characteristics of the study population, the anthropometric and biochemical measurements across glycemic conditions are summarized in Table 1. The median (inter-quartile range, IQR) of serum H_2_S concentrations across the glycemic statuses of the participants are reported in Table 1. Result of analysis of covariance showed no significant difference in serum H_2_S levels across the groups (*P* = 0.533).

**Table 1:** Characteristics of the study participants (*n* = 798)

	NFG-NGT (*n* = 511)	IIFG (*n* = 73)	IIGT (*n* = 65)	Combined IFG-IGT (*n* = 38)	T2DM (*n* = 111)
Age *(y)*	41.0 ± 12.6	47.1 ± 12.2	50.1 ± 14.1	55.8 ± 13.8	56.1 ± 12.5
Men *(%)*	46.6	50.7	47.7	50.0	53.2
FH *(%)*	7.4	12.5	7.8	10.8	16.2
BMI *(kg/m*^*2*^*)*	27.1 ± 5.0	30.4 ± 5.9	28.9 ± 4.9	29.8 ± 4.2	29.9 ± 4.8
WC *(cm)*	91.4 ± 11.7	99.3 ± 12.7	96.5 ± 10.8	100.0 ± 9.8	101.2 ± 11.2
SBP *(mm Hg)*	109 ± 13	118 ± 15	117 ± 18	121 ± 18	125 ± 17
DBP *(mm Hg)*	74 ± 9	79 ± 8	78 ± 11	75 ± 8	79 ± 8
FSG *(mg/dL)*	87.9 ± 6.5	104.4 ± 4.6	90.7 ± 5.1	107 ± 5.3	152.9 ± 59.5
2 h-SG *(mg/dL)*	98.9 ± 19.9	107.5 ± 18.5	156.4 ± 13.9	167.1 ± 15.7	243.7 ± 89.7^b^
TG *(mg/dL)*^a^	113 (80–159)	128 (98–194)	159 (123–204)	146 (120–213)	165 (118–224)
HDL-C *(mg/dL)*	48 ± 10	45 ± 11	44 ± 11	44 ± 11	43 ± 10
Serum H_2_S *(µmol/L)* ^a^	42.9 (22.2–81.0)	36.9 (17.4–88.1)	33.4 (19.3–70.9)	36.1 (9.3–81.1)	36.5 (18.9–71.1)

Data are mean ± SD (unless stated otherwise)

^a^Median (inter-quartile range)

^b^*n* = 32

*T2DM* type 2 diabetes mellitus, *FH* Family history of T2DM, *BMI* body mass index, *WC* waist circumference, *SBP* systolic blood pressure, *DBP* diastolic blood pressure, *FSG* fasting serum glucose, *2 h-SG* 2-h serum glucose, *TG* serum triglyceride, *HDL-C* high-density lipoprotein cholesterol, *H*_*2*_*S* hydrogen sulfide, *IFG* impaired glucose tolerance, *IGT* impaired glucose tolerance, *IIFG* isolated IFG, *IIGT* isolated IGT, *NFG-NGT* normal fasting glucose-normal glucose tolerance

The findings of multinomial logistic regression analyses are summarized in Table 2. Lower serum H_2_S (< 39.6 µmol/L) was associated with an increased chance of having IIGT in the adjusted model (OR = 1.96, 95% CI = 1.15–3.34). No significant association was observed between serum H_2_S and other dysglycemic conditions.

**Table 2:** The odds ratio (95% CI) of having dysglycemia according to serum H_2_S concentrations

	NGT-NFG	IIFG	IIGT	Combined IFG-IGT	T2DM
Log H_2_S					
*Crude*	1.00	0.93 (0.54–1.58)	0.67 (0.39–1.13)	0.58 (0.31–1.11)	0.80 (0.52–1.24)
*Adjusted model*	1.00	0.95 (0.55–1.62)	0.70 (0.41–1.20)	0.64 (0.33–1.24)	0.88 (0.55–1.40)
H_2_S (< median)					
*Case/total (n)*		38/73	41/65	21/38	60/111
*Crude*	1.00	1.24 (0.76–2.03)	1.96 (1.15–3.34)^*^	1.42 (0.73–2.75)	1.35 (0.89–2.04)
*Adjusted model*	1.00	1.27 (0.77–2.10)	1.97 (1.14–3.39)^**^	1.38 (0.69–2.74)	1.32 (0.84–2.06)

NGT-NFG was considered as the reference

Multinomial logistic regression was used (adjusted model included age and sex)

^*^P = 0.013, ^**^P = 0.014

Median serum H_2_S was 39.6 µmol*/L*

*T2DM* type 2 diabetes mellitus, *IFG* impaired fasting glucose, *IGT* impaired glucose tolerance, *IIFG* isolated IFG, *IIGT* isolated IGT

## Discussion

To the best of our knowledge, this is the first population-based study investigating the potential association between glycemic status and total serum sulfide levels, a surrogate of the novel gasotransmitter H_2_S, that is involved in glucose and insulin metabolism. Lower serum H_2_S (< 39.6 µmol/L) was associated with an increased chance of having IIGT, however other dysglycemic conditions were not related to serum H_2_S. This finding may imply that reduced H_2_S may be involved in the pathogenesis of T2DM via insulin resistance and glucose intolerance pathways.

Few studies often limited by lacking enough statistical power are available concerning endogenous H_2_S markers and glycemic parameters in humans. Median (IQR) plasma levels of H_2_S were reported to be lower in T2DM patients compared to lean-aged matched and obese subjects [10.5 (4.8–22.0) µmol/L *vs.* 38.9 (29.7–45.1) and 22.0 (18.6–26.7) µmol/L, respectively] [15]. Likewise, a lower serum level of H_2_S was detected in patients with T2DM compared with age-matched normal control subjects (110 *vs.* 130 µmol/L) [14]. Plasma H_2_S levels were significantly lower in patients with T2DM compared to healthy controls (45.1 ± 15.5 *vs.* 54.0 ± 26.4 µmol/L), and patients who had poor glycemic control had a more significant reduction in plasma H_2_S levels [25]. Plasma H_2_S levels were also negatively correlated with the glycosylated hemoglobin (HbA1c) level and disease duration [25].

Animal models of diabetes exhibited reduced serum H_2_S concentrations without changes in the tissue expression of the H_2_S synthesizing enzymes, cystathionine-β-synthase (CBS), and cystathionine-γ-lyase (CSE) [26]. These findings of in vivo studies suggest that hyperglycemia-induced H_2_S reduction may be due to increased degradation in response to high-glucose concentrations [26]. Furthermore, reduced activity of H_2_S-producing enzymes as seen in diabetes, reduces the production of endogenous H_2_S and its circulatory levels [27]. Hydrogen sulfide deficiency has been suggested to be involved in the progression of diabetes and its complications [28].

In mammals, circulating H_2_S concentrations have been reported to range from nanomolar to micromolar levels [29]. Physiological serum H_2_S concentrations appear within a range of 30–300 µmol/L [30]. As with other gasotransmitters, i.e., nitric oxide (NO) and carbon monoxide (CO), biological effects of H_2_S follow a biphasic dose–response, including physiological and cytoprotective properties at low concentrations to cytotoxic effects at higher concentrations [31].

Our study has some limitations. Due to its cross-sectional design, it is difficult to make a causal inference between serum H_2_S and dysglycemia, and the associations identified might be challenging to interpret. Another limitation was the method used to measure serum total sulfide level, the methylene blue method, which measures all sulfur species rather than only free-H_2_S. Interference of other colored substances, formation of methylene blue dimer and trimer, strong acid chemical pretreatment, and low sensitivity have been documented as limitations of the method [20]. However, this method is commonly used for measuring H_2_S in biological systems. As an strength, our findings may provide new insights into further investigations.

In conclusion, the findings of this cross-sectional study indicate that reduced serum H_2_S concentrations may be associated with an impaired glucose tolerance. This may imply that H_2_S deficiency is probably involved in or H_2_S system is down-regulated during the progression of T2DM.

## Supplementary Information

**Additional file 1: Figure 1.** Standard calibration curve of serum H_2_S measurement.

## Data Availability

Data will be presented upon forwarding the request to the corresponding author (ghasemi@endocrine.ac.ir) and confirmation of the director of RIES (azizi@endocrine.ac.ir).

## Abbreviations

2 h-SG: 2 hours serum glucose
BMI: Body mass index
DBP: Diastolic blood pressure
FSG: Fasting serum glucose
H_2_S: Hydrogen sulfide
HDL-C: High-density lipoprotein cholesterol
IFG: Impaired fasting glucose
IGT: Impaired glucose tolerance
IQR: Inter-quartile range
IIFG: Isolated impaired fasting glucose
IIGT: Isolated impaired glucose tolerance
NFG: Normal fasting glucose
NGT: Normal glucose tolerance
OGTT: Oral glucose tolerance test
OR: Odds ratio
SBP: Systolic blood pressure
SD: Standard deviation
T2DM: Type 2 diabetes mellitus
TG: Triglyceride
TLGS: Tehran Lipid and Glucose Study
WC: Waist circumference
