# Supplementary material for: Association between serum hydrogen sulfide concentrations and dysglycemia: a population-based study
Source: BMC Endocr Disord. 2022 Mar 28;22:79. doi: 10.1186/s12902-022-00995-8 (PMC8962595; doi:10.1186/s12902-022-00995-8)
Supplement: Supplementary file 1 — Additional file 1: Figure 1. Standard calibration curve of serum H2S measurement. [file 12902_2022_995_MOESM1_ESM.docx]

**Supplementary Figure 1.** Standard calibration curve of serum H_2_S measurement
